# Trait reward sensitivity and behavioral motivation shape connectivity between the default mode network and the striatum during reward anticipation

**DOI:** 10.1101/2025.04.17.649386

**Published:** 2025-05-07

**Authors:** James B. Wyngaarden, Akanksha Nambiar, Jeffrey Dennison, Lauren B. Alloy, Dominic S. Fareri, Johanna M. Jarcho, David V. Smith

**Affiliations:** 1. Temple University; 2. University of West Bohemia; 3. University of Pennsylvania; 4. Adelphi University

## Abstract

Individuals vary substantially in their responses to rewarding events and their motivation to pursue rewards. The ventral striatum (VS) plays a key role in reward anticipation, and connectivity between the VS and the default mode network (DMN)—a network associated with self-referential and evaluative processes—has been implicated in reward processing. However, the relationship between these neural mechanisms and reward-related individual differences remains unclear. In the present study, we examined how trait reward sensitivity and behavioral motivation shape connectivity between the default mode network (DMN) and the ventral striatum (VS) during reward anticipation. Forty-six participants completed the Monetary Incentive Delay (MID) task while undergoing fMRI, with trial types reflecting varying levels of reward and loss salience. Behavioral measures of motivation were derived from reaction time contrasts between large and neutral trials, and self-reported anhedonia and reward sensitivity were assessed. We found that individuals with higher reward sensitivity exhibited greater striatal connectivity with DMN during reward-salient trials, highlighting the VS’s role in incentive processing. However, this relationship was moderated by behavioral motivation. Specifically, in individuals with high behavioral motivation, reward sensitivity was associated with reduced DMN-VS connectivity during reward anticipation. In contrast, for those with lower behavioral motivation, the relationship between reward sensitivity and DMN-VS connectivity was attenuated. These results provide novel insights into the neural correlates of individual differences in reward processing, demonstrating that behavioral motivation is crucial in understanding DMN-striatal interactions during reward anticipation. These findings highlight the importance of considering motivational context when investigating reward-related neural mechanisms.

## Introduction

Reward sensitivity is a fundamental psychological construct that explains individual variations in motivational responsiveness and goal-directed behavior (Gray, 1987; Fellows, 2004). This trait describes how individuals perceive, process, and engage with potential rewards (Capa & Bouquet, 2018; Veldhoven et al., 2020). It is associated with increased responding from reward-related brain regions, including the ventral striatum (VS), ventromedial prefrontal cortex (vmPFC), and orbitofrontal cortex (OFC) (Volkow et al., 2002; Carter et al., 2009; Haber & Knutson, 2010). Reward sensitivity critically shapes behavioral outcomes across different contexts. Heightened reward sensitivity correlates with increased goal persistence but also predisposes individuals to impulsive behavior characteristic of addiction and compulsive gambling (Davis et al., 2007; Gaher et al., 2015). Conversely, diminished reward sensitivity—typically manifested as anhedonia, a reduced capacity to experience pleasure—is a key clinical feature in depressive and affective disorders (Alloy et al., 2016). These contrasting neuropsychological presentations highlight reward sensitivity as a transdiagnostic factor with significant implications for understanding behavioral regulation and mental health (Potsch & Rief, 2023; Oka et al., 2024). Investigating the complex interactions between reward sensitivity and contextual motivational factors is essential for comprehending the mechanisms underlying reward-seeking behavior.

Behavioral motivation represents the manifestation of one’s internal predisposition of reward sensitivity into observable action, serving as a critical interface between individual psychological traits and environmental demands (Davis et al., 2007). Unlike reward sensitivity, which represents a relatively stable trait (Jorm et al., 1998; Cardoso Melo et al., 2022; Cardoso Melo et al., 2023), behavioral motivation refers to a dynamic and context-dependent state that is modulated by the perceived salience of potential rewards and losses (Hull, 1943; Duffy, 1957). Incentive salience, the motivational significance attributed to rewards and losses (Berridge & Robinson, 1998), drives both neural processing and behavioral responses. In this study, we measured behavioral motivation—the behavioral expression of incentive salience—using reaction time (RT) patterns in the Monetary Incentive Delay (MID) task, indexing it through three contrasts: HS>LS (high stakes vs. low stakes), which captures the overall pattern of faster RTs for high-stakes trials (large gains/losses) compared to low-stakes trials (neutral/small gains/losses); LG>N (large gain vs. neutral), reflecting motivation for large rewards; and LL>N (large loss vs. neutral), reflecting motivation to avoid significant losses. Empirical observations indicate that tasks involving high stakes rewards typically elicit more intense motivational responses, particularly among individuals with elevated reward sensitivity (Hernandez Lallement et al., 2014; Chat et al., 2022). Although some trait-level measures, such as the Behavioral Activation Scale (BAS; Carver & White, 1994) and Sensitivity to Punishment Sensitivity to Reward Questionnaire (SPSRQ; Torrubia et al., 2001), capture broad tendencies toward reward-driven behavior, motivation in real-world contexts often depends on how individuals allocate effort in response to specific incentives. However, this relationship is not straightforward; complex contextual factors and individual variability significantly moderate how trait reward sensitivity translates into motivational behavior (Rosell-Negre et al., 2017; Goldstein et al., 2007). Despite extensive research on both of these psychological constructs, empirical research directly examining their joint influence on reward anticipation remains limited. Addressing this research gap requires a nuanced investigation of how behavioral motivation and reward sensitivity dynamically interact to shape reward processing and behavioral outcomes.

The relationship between reward sensitivity and behavioral motivation is further clarified by examining anticipatory pleasure. The Temporal Experience of Pleasure Scale (TEPS) specifically assesses anticipatory pleasure, a key component of anhedonia that relates to the experience of “wanting” versus “liking” rewards (Gard et al., 2006). Anticipatory pleasure is linked to motivation and goal directed behavior, which leads one to having the experience of wanting more. This conceptual framework provides an integrated perspective on how reward sensitivity, behavioral motivation, and anticipatory pleasure interact within reward processing networks, helping to disambiguate the neural mechanisms of approach motivation from hedonic experience.

The ventral striatum (VS) plays a pivotal role in reward anticipation, serving as a core structure for processing incentive salience and value (Filimon et al., 2020). Activation of the VS during reward anticipation reflects its importance in translating reward-related cues into behavioral responses (Bretzke et al., 2021; Carruzzo et al., 2023). Beyond local activation, connectivity between the VS and broader networks, like the default mode network (DMN), has emerged as a critical area of inquiry. Although reward sensitivity shapes behavior, its influence on large-scale neural connectivity, particularly between the VS and DMN, remains less understood. The DMN, traditionally associated with self-referential and evaluative processes, increasingly is recognized for its role in integrating motivational and evaluative signals during reward anticipation. Recent studies suggest that VS-DMN connectivity may reveal how individual differences in reward sensitivity and motivation shape neural responses (Dobryakova & Smith, 2021; Grill et al., 2021), though findings are inconsistent. Some evidence links higher reward sensitivity to increased VS-DMN connectivity. For example, aberrant reward sensitivity has been associated with increased functional connectivity between the VS and vmPFC during social rewards (Wyngaarden et al., 2024), and similar alterations in DMN connectivity have been observed in individuals at high risk for depression (Posner et al., 2016), suggesting complex neural mechanisms underlying reward processing and affective states. In contrast, other studies suggest attenuation of connectivity under high motivational demand. For example, under high motivational demands, reward anticipation tasks reveal decreased network segregation and reduced dissociation between attentional and valuation networks, with notable implications for social dysfunction and reward processing (Pessoa, 2015; Saris et al., 2020). These discrepancies point to a critical gap in understanding: how do individual differences in reward sensitivity and motivation jointly influence VS activation and its connectivity with the DMN during reward anticipation?

To address these gaps, the current study investigates the relationships between trait reward sensitivity, state-dependent behavioral motivation, and VS-DMN connectivity during reward anticipation. Using an fMRI-based Monetary Incentive Delay (MID) task, we examine how trait reward sensitivity and task-driven motivation influence neural responses across varying levels of reward and loss salience. We hypothesize that reward sensitivity will predict distinct patterns of VS activation and DMN-VS connectivity, with behavioral motivation moderating these relationships. Specifically, we anticipate that reward sensitivity will correlate with greater VS activation and altered connectivity with the DMN, particularly in motivationally salient contexts. By integrating behavioral and neural perspectives, this study aims to provide a comprehensive understanding of how reward-related traits and states shape the neural mechanisms underlying reward anticipation.

## Materials and Methods

### Participants

This dataset is available as OpenNeuro Dataset 4920 (Smith et al., 2024), and it is composed of neuroimaging data from 59 participants who completed four tasks involving social and nonsocial reward processing. The data are organized in accordance with the Brain Imaging Data Structure (BIDS) specification (K. J. Gorgolewski et al., 2016) using HeuDiConv (Halchenko et al., 2024). The pre-registration (https://aspredicted.org/PQA_WPB) describes the goal to collect data from 100 participants (18–22), wherein we acquired data from 60 participants due to constraints imposed by the COVID-19 pandemic. As per pre-registered criteria, fourteen of the 60 participants who completed the study were excluded from analyses due to their failure to respond during behavioral tasks (>20% missing responses; N=4), incomplete data (N=4; failure to complete survey data or missing behavioral data due to technical issues), or poor image quality (N=6). Image quality was defined using the fd_mean and tSNR values from MRIQC. Participants were excluded for fd_mean values greater than 1.5 times the interquartile range, per the distribution from neuroimaging data of otherwise eligible participants. This resulted in the final set of 48 participants (mean age: 20.45 yrs, SD: 1.89 yrs; 22.7% male of which 57% white, 34% Asian, 9% other- 2 Black/African American, 1 black and white, 1 Indian).

Participants were recruited via the Temple University Psychology and Neuroscience Department participant pool, and from the surrounding community via flyers and online advertisements. Participants were paid $25 per hour for fMRI and $15 per hour for behavioral tasks, and received bonuses based on their decisions on other neuroeconomic tasks (not reported here), resulting in a total payment of $140 to $155. In addition, participants recruited from the university pool also received research credit for their participation.

### Procedure

All methods were approved by the Temple University IRB. Prospective participants were identified based on their responses to an online screener questionnaire, which assessed RS using the Behavioral Activation Subscale (BAS; Carver & White, 1994) and the Sensitivity to Reward subscale (SR; Torrubia et al., 2001). A sum was calculated for each subscale. Sums were assigned to a quintile that reflected low to high levels of RS across the distribution of possible scores. We used methods consistent with our prior work (e.g., Alloy et al., 2012) to ensure participants were responding truthfully and attentively. Only participants with scores within +/−1 quintile on both subscales were eligible for the study (no exclusions were made based on this criteria). At the in-person visit, we confirmed that eligible participants were free of major psychiatric or neurologic illness and MRI contraindications. Prior to MRI scanning, all participants underwent safety screening including verification of MRI compatibility to ensure data quality and participant safety.

#### fMRI-based Monetary Incentive Delay Task

**Figure 2.**
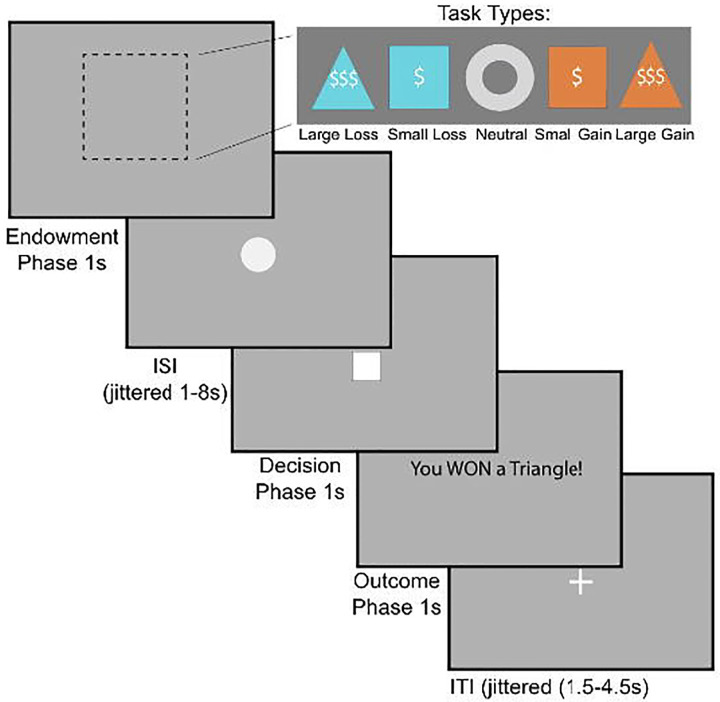
The monetary incentive delay (MID) task. During the endowment phase, participants see a cue indicating the trial type: large gain, small gain, large loss, small loss, or neutral (1s). Then, after a jittered interval (1–8s), the target square appears (1s), during which time participants must respond with a button press. This is followed by an outcome phase (1s). Trials are separated by a jittered interval (1.5–4.5s). Figure adapted from Smith et al., 2024 with permission.

In order to probe behavioral motivation participants were subjected to the Monetary Incentive Delay Task (MID) (Knutson et al., 2000). During the task participants respond to a stimulus in order to either gain money or avoid losing money (Fig. 2). There are 5 conditions, corresponding with the 5 different shapes in the top panel (Large Loss, Small Loss, Neutral, Small Gain, Large Gain). Precisely, the value of money presented on the screen was (-$5, +$1, $0, -$1, +$5). During the endowment phase, participants see a shape which indicates the money at stake for the current trial. They are then presented with an ISI (inter-stimulus interval) period until a white square appears. Once the square appears, they have 1 second to respond. If they respond in time, they win the gain trials, acquiring money, and in loss trials, they avoid losing money. In contrast, if they do not respond quickly, they lose the trial; i.e. in gain trials they don’t win money, and in loss trials they lose money. Thus, the MID task analyzes people’s behavior based on reaction time or how quickly people respond when the cue appears.

#### Individual difference measures

Reward sensitivity (RS) was assessed using a composite measure derived from the z-scores of two self-report scales: the Behavioral Activation System (BAS) scale (Carver & White, 1994) and the reward subscale of the Sensitivity to Punishment and Sensitivity to Reward Questionnaire (SPSRQ; Torrubia et al., 2001). The BAS scale is a 20-item questionnaire designed to measure individual differences in sensitivity to reward and approach motivation. The SPSRQ reward subscale consists of 24 items assessing sensitivity to reward in specific contexts. Both the BAS and SPSRQ reward subscale have been established as reliable and valid measures of trait reward sensitivity (Alloy et al., 2006; Alloy et al., 2012). Additionally, we included the Temporal Experience of Pleasure Scale (TEPS; Gard et al., 2006), an 18-item measure designed to assess distinct components of pleasure experience. Given that the monetary incentive delay task used in this study focuses on reward anticipation, we specifically analyzed the anticipatory subscale of the TEPS.

**Figure 3.**
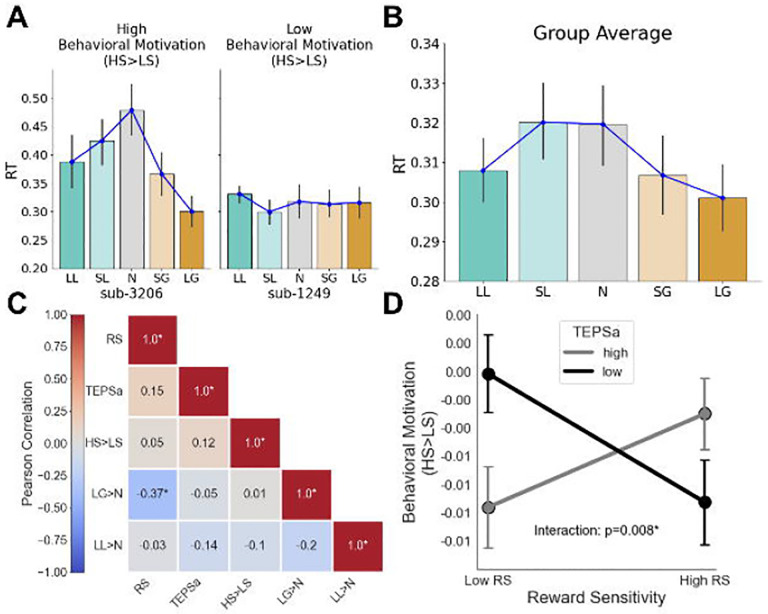
Self-reported anhedonia (TEPSa) modulates the relationship between Reward Sensitivity (RS) and Behavioral Motivation (HS>LS). (**A**) Examples of high (characterized by faster RTs for high stakes trials relative to low stakes trials) vs. low (no difference in RT between trials) behavioral motivation. (**B**) Behavioral motivation across the sample. On average, participants responded faster for large gain and loss trials than for neutral trials, demonstrating behavioral motivation on reward-salient trials. (**C**) Correlation heatmap of variables of interest, where LG>N refers to the contrast in RTs for Large Gains vs. Neutral and LL>N refers to the contrast in RTs for Large Losses vs. Neutral. (**D**) Interaction plot depicting the relationship between behavioral motivation (HS>LS), self-reported anhedonia (TEPSa), and reward sensitivity, showing predicted motivation levels across low and high reward sensitivity (10th/90th percentiles) for low and high TEPSa groups (median-split), with standard error of the mean (SEM) error bars and a significant interaction (p < 0.01, annotated). For individuals with higher reward sensitivity, high anticipatory pleasure is associated with greater behavioral motivation relative to low anticipatory pleasure, an effect that flips for individuals with lower reward sensitivity.

We also aimed to examine individual differences in behavioral motivation during the MID task. We operationalized behavioral motivation as the extent to which reaction times (RTs) were influenced by reward salience, indexing it through three RT contrasts (Fig. 3A). First, HS>LS (high stakes vs. low stakes) captures the overall pattern of RT differentiation across trial types, such that individuals with greater behavioral motivation responded more quickly to high-stakes trials (large gain: +$5; large loss: -$5) compared to low-stakes trials (neutral: $0; small gain: +$1; small loss: -$1). To quantify this, we fitted a second-degree polynomial to each participant’s RTs across all five trial types, extracting the quadratic coefficient as HS>LS. A more negative HS>LS value indicates a stronger pattern of faster RTs for high-stakes trials and slower RTs for low-stakes trials, reflecting greater behavioral motivation, while a less negative or near-zero value indicates weaker RT differentiation, reflecting lower motivation. Second, we examined specific contrasts: LG>N (large gain vs. neutral) captures motivational responses to large rewards, and LL>N (large loss vs. neutral) captures motivation to avoid significant losses. Notably, avoiding a large loss is itself a highly motivating outcome, as individuals often treat potential losses as psychologically equivalent to missing out on a comparable gain (Kahneman & Tversky, 1979). This aligns with prior evidence that loss aversion engages reward-processing circuitry, reinforcing the idea that both large gains and large losses can drive motivated behavior. These complementary approaches allowed us to assess both the overall pattern of behavioral motivation (via HS>LS) and its sensitivity to specific reward or loss conditions (via LG>N and LL>N).

#### Correction for Multiple Comparisons

For analyses involving pairwise comparisons between trial conditions, we conducted a total of 10 tests (comparing each of the five conditions—Large Gain, Small Gain, Neutral, Small Loss, and Large Loss—against every other condition). To control for the family-wise error rate and minimize Type I errors, we applied Tukey’s Honest Significant Difference (HSD) correction for all pairwise post-hoc comparisons following significant omnibus ANOVA results. For our exploratory analyses examining the relationships between individual difference measures (reward sensitivity, anhedonia) and neural responses, we report both uncorrected p-values and whether findings survived correction for multiple comparisons. Specifically, for analyses investigating VS activation across different reward contrasts, we considered a family of 3 tests (incentive salience contrasts, HS>LS, LG>N, LL>N) and applied a Bonferroni-corrected significance threshold of p < 0.0167 (0.05/3). Similarly, for our analyses of DMN-VS connectivity, we considered the same family of 3 contrasts and interaction terms with individual difference measures, applying the same correction threshold. All statistical analyses were performed using R (version 4.0.3; R Core Team, 2020), and results that did not survive multiple comparison correction are explicitly noted as exploratory findings.

#### Neuroimaging Data Acquisition

These data were collected as part of an overarching project that has been described in a previous publication (for full details see Smith et al., 2024). Images were acquired using a simultaneous multi-slice (multi-band factor = 2) gradient echo-planar imaging (EPI) sequence (240 mm in FOV, TR = 1,750 ms, TE = 29 ms, voxel size of 3.0 × 3.0 × 3.0 mm^3^, flip angle = 74, interleaved slice acquisition, with 52 axial slices). Each run included 292 functional volumes. We also collected single-band reference images with each functional run of multi-band data to improve motion correction and registration. To facilitate anatomical localization and co-registration of functional data, a high-resolution structural scan was acquired (sagittal plane) with a T1-weighted magnetization=prepared rapid acquisition gradient echo (MPRAGE) sequence (224 mm in FOV, TR = 2,400 ms, TE = 2.17 ms, voxel size of 1.0 × 1.0 × 1.0 mm^3^, flip angle 8°). In addition, we also collected a B0 fieldmap to unwarp and undistort functional images (TR: 645 ms; TE1: 4.92 ms; TE2: 7.38 ms; matrix 74×74; voxel size: 2.97×2.97×2.80 mm^3^; 58 slices, with 15% gap; flip angle: 60°)

#### Pre-processing of Neuroimaging Data

Neuroimaging data were converted to the Brain Imaging Data Structure (BIDS) using HeuDiConv (Halchenko et al., 2024). Results included in this manuscript come from pre-processing performed using fMRIPrep 20.2.3 (Esteban et al., 2019), which is based on Nipype 1.4.2 (Gorgolewski et al., 2011, 2016).

##### Anatomical data pre-processing

The T1-weighted (T1w) image was corrected for intensity non-uniformity (INU) with `N4BiasFieldCorrection`, distributed with ANTs 2.3.3, and used as T1w-reference throughout the workflow. The T1w-reference was then skull-stripped with a *Nipype* implementation of the `antsBrainExtraction.sh` workflow (from ANTs), using OASIS30ANTs as target template. Brain tissue segmentation of cerebrospinal fluid (CSF), white-matter (WM), and gray-matter (GM) was performed on the brain-extracted T1w using `fast` (FSL 5.0.9). Volume-based spatial normalization to one standard space (MNI152NLin2009cAsym) was performed through nonlinear registration with `antsRegistration` (ANTs 2.3.3), using brain-extracted versions of both T1w reference and the T1w template. The following template was selected for spatial normalization: *ICBM 152 Nonlinear Asymmetrical template version 2009c* (TemplateFlow ID: MNI152NLin2009cAsym)

##### Functional data pre-processing

Primarily, for each of the BOLD runs per subject, the following pre-processing steps were performed. First, a reference volume and its skull-stripped version were generated by aligning and averaging 1 single-band references (SBRefs). A B0- nonuniformity map (or *field map*) was estimated based on a phase-difference map calculated with a dual-echo GRE (gradient-recall echo) sequence, processed with a custom workflow of *SDCFlows* inspired by the `epidewarp.fsl` script, (http://www.nmr.mgh.harvard.edu/~greve/fbirn/b0/epidewarp.fsl) and further improvements in HCP Pipelines. The *field map* then was co-registered to the target EPI (echo-planar imaging) reference run and converted to a displacements field map (amenable to registration tools such as ANTs) with FSL’s `fugue` and other *SDCflows* tools. Based on the estimated susceptibility distortion, a corrected EPI (echo-planar imaging) reference was calculated for a more accurate co-registration with the anatomical reference. The BOLD reference was then co-registered to the T1w reference using `flirt` (FSL 5.0.9) with the boundary-based registration cost-function. Co-registration was configured with nine degrees of freedom to account for distortions remaining in the BOLD reference. Head-motion parameters with respect to the BOLD reference (transformation matrices, and six corresponding rotation and translation parameters) are estimated before any spatiotemporal filtering using `mcflirt`. Further, we applied spatial smoothing with a 5mm full-width at half-maximum (FWHM) Gaussian kernel using FEAT (FMRI Expert Analysis Tool) Version 6.00, part of FSL (FMRIB’s Software Library, www.fmrib.ox.ac.uk/fsl). Non-brain removal using BET (Smith, 2002) and grand mean intensity normalization of the entire 4D dataset by a single multiplicative factor were also applied.

BOLD runs were slice-time corrected using `3dTshift` from AFNI 20160207. First, a reference volume and its skull-stripped version were generated using a custom methodology of *fMRIPrep*. The BOLD time-series (including slice-timing correction when applied) were resampled onto their original, native space by applying a single, composite transform to correct for head-motion and susceptibility distortions. These resampled BOLD time-series will be referred to as *preprocessed BOLD in original space*, or just *preprocessed BOLD*. The BOLD time-series were resampled into standard space, generating a *preprocessed BOLD run in MNI152NLin2009cAsym space*. First, a reference volume and its skull-stripped version were generated using a custom methodology of *fMRIPrep*. Several confounding time-series were calculated based on the *preprocessed BOLD*, notably including framewise displacement (FD).

Additionally, a set of physiological regressors were extracted to allow for component-based noise correction (*CompCor*). These components are estimated after high-pass filtering; the *preprocessed BOLD* time-series (using a discrete cosine filter with 128s cut-off) for anatomical component correction (aCompCor). For aCompCor, three probabilistic masks (CSF, WM and combined CSF+WM) are generated in anatomical space. The implementation differs from that of Behzadi et al. in that instead of eroding the masks by 2 pixels on BOLD space, the aCompCor masks are subtracted from a mask of pixels that likely contain a volume fraction of GM. This mask is obtained by thresholding the corresponding partial volume map at 0.05, and it ensures components are not extracted from voxels containing a minimal fraction of GM. Finally, these masks are resampled into BOLD space and binarized by thresholding at 0.99 (as in the original implementation). Components are also calculated separately within the WM and CSF masks. For each CompCor decomposition, the *k* components with the largest singular values are retained, such that the retained components’ time series are sufficient to explain 50 percent of variance across the nuisance mask (CSF, WM, combined, or temporal). The remaining components are dropped from consideration. The head-motion estimates calculated in the correction step also were placed within the corresponding confounds file. All resamplings can be performed with a single interpolation step by composing all the pertinent transformations (i.e., head-motion transform matrices, susceptibility distortion correction when available, and co-registrations to anatomical and output spaces). Gridded (volumetric) resamplings were performed using `antsApplyTransforms` (ANTs), configured with Lanczos interpolation to minimize the smoothing effects of other kernels.

Many internal operations of *fMRIPrep* use *Nilearn* 0.6.2, mostly within the functional processing workflow. For more details of the pipeline, see the section corresponding to workflows in *fMRIPrep*’s documentation (https://fmriprep.readthedocs.io/en/latest/workflows.html).

#### FMRI Analyses

##### Individual level analyses

Neuroimaging analyses used FSL version 6.0.0 (Smith et al., 2004; Jenkinson et al., 2012). We conducted two types of analyses (activation and network connectivity) to investigate how incentive salience, behavioral motivation, and trait reward sensitivity were associated with BOLD responses. Both used individual-level general linear models with local autocorrelation (Woolrich et al., 2001).

The first model focused on the brain activation evoked during the anticipation phase and used seven task-based regressors, including anticipation of large gains, small gains, large losses, small losses, neutral trials, hits, and misses (jittered = 1000–8000 ms). Notably, this anticipation-focused model allowed us to isolate neural activity associated with reward anticipation rather than reward outcome. Motion parameters derived from realignment were included as covariates of no interest to control for movement-related artifacts and provides a robust framework for examining how reward anticipation recruits striatal and default mode network regions.

The second model focused on task-dependent connectivity with the Default Mode Network (DMN) as it related to the varying magnitudes of reward (Utevsky et al., 2017; Fareri et al., 2021; Smith et al., 2014). To estimate the changes in connectivity between feedback types, we used network psychophysiological interaction (nPPI) analysis (e.g., Friston et al., 1997; O’Reilly et al., 2012), which can reveal consistent and specific task-dependent changes in connectivity (Smith, 2016; Smith & Delgado, 2017). We focused on incentive-salience-dependent changes in DMN connectivity with the bilateral VS (defined by the Oxford-GSK-Imanova atlas; Tziortzi et al., 2011). The first seven regressors in this model were identical to those described in the activation model (i.e. reward magnitude, hits, and misses). The DMN and nine additional networks, including the executive control network (ECN), were defined based on prior work (Smith et al., 2009). Network time courses were extracted with a spatial regression component of the dual regression approach (Nickerson et al., 2017) and included as ten additional regressors in the model. Finally, PPI regressors were formed by multiplying each of the seven task regressors by the DMN regressor, yielding a total of 24 regressors.

Both activation and connectivity models included additional regressors of no interest that controlled for six motion parameters (rotations and translations), the first six aCompCor components explaining the most variance, non-steady state volumes, and framewise displacement (FD) across time. Finally, high-pass filtering (128s cut-off) was achieved using a set of discrete cosine basis functions.

##### ROI-based group-level activation analyses

Our first hypothesis seeks to test whether individuals demonstrating higher levels of reward sensitivity will show exaggerated VS activation for incentive-salient trials (Cooper & Knutson, 2008). Given prior evidence linking anhedonia to altered reward processing (Daniels et al., 2025), we included TEPSa to examine whether anhedonia moderates corticostriatal connectivity patterns (Haber & Knutson, 2010). For each participant, activation in the VS seed described above was extracted using fslmeants. We then conducted a linear regression for the difference in extracted striatal BOLD for task-based contrasts targeting incentive salience [i.e., high stakes > low stakes (HS>LS), large gain>neutral (LG>N), and large loss>neutral (LL>N)] that was regressed onto a model of composite reward sensitivity (RS) and self-reported anhedonia (TEPSa) and their interaction (RS x TEPSa).

##### ROI-based group-level nPPI analyses

We also examined whether reward hyposensitive individuals exhibit enhanced corticostriatal connectivity with the default mode network (DMN) compared to individuals with more moderate reward sensitivity. Fundamentally, we used nPPI to account for connectivity across multiple networks, allowing us to capture broader patterns of functional coupling beyond pairwise interactions. We hypothesized a negative relationship between reward sensitivity and corticostriatal connectivity with DMN, such that reward-hyposensitive individuals will exhibit enhanced connectivity and reward-hypersensitive individuals will exhibit blunted connectivity. We further hypothesized that both enhanced/blunted corticostriatal connectivity with DMN would be associated more strongly with behavioral motivation than hedonic anhedonia. To test these hypotheses, we conducted an ROI-based network psychophysiological interaction (nPPI) analysis using the DMN as the seed and VS as the target. This seed was chosen because the DMN is a higher-order network implicated in self-referential processing and value-based decision-making, while the VS plays a central role in reward processing and motivation. Using the DMN as the seed allows us to examine how individual differences in reward sensitivity are related to variations in corticostriatal connectivity during reward anticipation, without making assumptions about the causal direction of these relationships. Connectivity estimates for VS were extracted using fslmeants from individual level analyses that modelled the time course of DMN as a seed. We then conducted linear regressions for the difference in connectivity for task-based contrasts targeting incentive salience [i.e., high stakes > low stakes (HS>LS), large gain>neutral (LG>N), and large loss>neutral (LL>N)], regressing these contrasts onto models of composite reward sensitivity (RS), Behavioral Motivation (matching the RT contrast to the PPI contrast), and their interaction (RS x Behavioral Motivation). We also tested models of RS x TEPSa and Behavioral Motivation x TEPSa. Given prior evidence linking anhedonia to altered reward processing, we included TEPSa to examine whether anhedonia moderates corticostriatal connectivity patterns

## Results

### Behavioral motivation and individual differences

Our first aim was to examine the relationships between behavioral motivation, self-reported anhedonia (TEPS), and reward sensitivity during reward anticipation. Behavioral motivation, a dynamic state reflecting the drive to act based on incentive salience, was indexed by reaction time (RT) contrasts in the Monetary Incentive Delay (MID) task (see Methods). Figure 3A illustrates this concept using HS>LS (high stakes vs. low stakes), which captures the overall pattern of RT differentiation: faster responses to high-stakes trials (large gains or losses) compared to low-stakes trials (neutral or smaller-stake trials) indicate higher behavioral motivation, while flatter RT patterns reflect lower motivation.

This pattern was evident at the group level (Fig. 3B). A repeated-measures ANOVA revealed a significant effect of condition on RTs, *F*(4,180)=5.022, *p*<0.001. Post-hoc pairwise comparisons using Tukey’s HSD confirmed that participants responded faster to incentive-salient trials: RTs in the Large Gain condition were significantly lower than those in the Neutral (*t*=−3.525, *p*=0.0048) and Small Loss conditions (*t*=−2.768, *p*=0.0484), while Neutral RTs were significantly higher than those in the Large Loss condition (*t*=3.454, *p*=0.0061). No other pairwise comparisons reached statistical significance. Full pairwise comparisons across trial conditions are available in Supplementary Table 1. These findings demonstrate that, on average, participants exhibited greater behavioral motivation for large gain and loss trials compared to neutral trials, consistent with the motivational salience of high-stakes incentives.

We next explored how behavioral motivation (HS>LS) relates to individual differences in reward sensitivity (RS) and self-reported anhedonia (TEPSa). Figure 3C presents a correlation heatmap of these variables, including specific RT contrasts large gain > neutral (LG>N) and large loss > neutral (LL>N). Contrary to our hypothesis, HS>LS was not significantly correlated with either self-reported anhedonia (*r*=0.12, *p*=0.43) or reward sensitivity (*r*=0.052, *p*=0.73). However, a significant negative correlation emerged between LG>N and reward sensitivity, indicating that individuals with greater reward sensitivity exhibited faster reaction times (RTs) for large gain trials compared to neutral trials. Moreover, we identified a significant interaction between reward sensitivity and anhedonia in predicting HS>LS (*t*(46)=2.799, *p*=0.00778; Fig. 3D). At lower levels of anhedonia (i.e., high TEPSa), higher reward sensitivity was associated with a more pronounced HS>LS pattern, reflecting enhanced behavioral motivation. In contrast, at higher levels of anhedonia, the relationship between reward sensitivity and HS>LS flips, suggesting that anhedonia moderates how trait-level reward sensitivity translates into motivated behavior.

### Reward sensitivity and striatal responses

**Figure 4.**
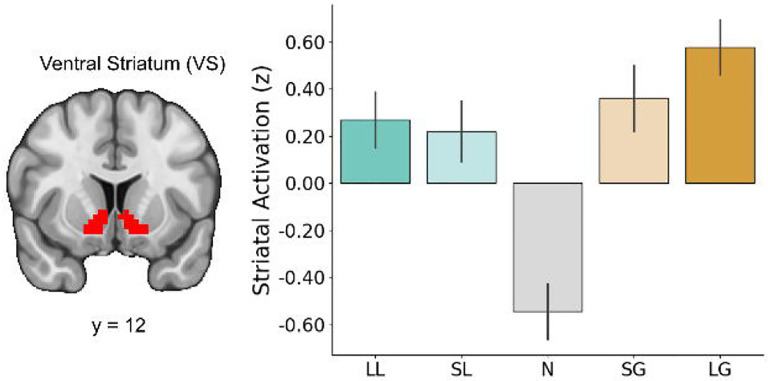
Striatal response is sensitive to incentive magnitude. Signal from the ventral striatum (VS) during the anticipation phase shows heightened activation during incentive-salient trials relative to neutral trials.

We also hypothesized that reward hyposensitive individuals would exhibit blunted striatal responses to reward anticipation and that reward hypersensitive individuals would exhibit enhanced striatal responses to reward anticipation, relative to individuals with moderate reward sensitivity. To assess this hypothesis, we first extracted signal from the VS across all trial types (Fig. 4). A repeated-measures ANOVA revealed a significant main effect of condition on the measured response, *F*(4, 180)=30.84, *p*<0.001. Post-hoc pairwise comparisons using Tukey’s HSD indicated several significant differences among conditions. Striatal responses in the Large Gain condition were significantly higher than those in the Neutral condition (*t=10.348, p*<0.0001), the Small Loss condition (*t*=3.288, *p*=0.0105), and the Large Loss condition (*t*=2.845, *p*=0.0393). Similarly, responses in the Small Gain condition were significantly higher than those in the Neutral condition (*t*=8.351, *p*<0.0001). The Neutral condition elicited significantly lower responses compared to both the Small Loss condition (*t*=−7.060, *p*<0.0001) and the Large Loss condition (*t*=−7.503, *p*<0.0001). No significant differences were observed between the Small Gain, Small Loss, and Large Loss conditions. Full pairwise comparisons across trial conditions are available in Table 2.

We next examined whether reward sensitivity (RS) and self-reported anhedonia (TEPSa) were associated with striatal response to varying trial types. We hypothesized that aberrant striatal responses to reward anticipation will be associated more strongly with the self-reported anhedonia (i.e., TEPSa) than hedonic anhedonia (i.e., a deficit in experiencing pleasure). To test these hypotheses, we conducted linear regressions for the difference in striatal BOLD for task-based contrasts targeting incentive salience (i.e., HS>LS, LG>N, LL>N) that was regressed onto a model of composite reward sensitivity (RS) and self-reported anhedonia (TEPSa) and their interaction (RS x TEPSa). Exploratory analyses included additional covariates for behavioral motivation.

We found no significant interaction between reward sensitivity and anhedonia in relation to striatal response for HS>LS (*t*=0.517, *p*=0.608), LG>N (*t*=0.923, *p*=0.361), or LL>N (*t*=−0.869, *p*=0.390). Exploratory analyses of lower order effects also did reveal that aberrant reward sensitivity (i.e., reward hypo- and hyper-sensitive individuals) was associated with striatal response to large vs. small gains, such that aberrant reward sensitivity was associated with reduced striatal activation relative to moderate reward sensitivity (*t*=2.368, *p*=0.023), although these did not survive correction for multiple comparisons. Full comparisons are available in Supplementary Table 2.

### Corticostriatal connectivity with Default Mode Network (DMN)

Our final analyses examined whether reward hyposensitive individuals exhibit enhanced corticostriatal connectivity with the default mode network (DMN) compared to individuals with more moderate reward sensitivity. We hypothesized a negative relationship between reward sensitivity and corticostriatal connectivity with DMN, such that reward-hyposensitive individuals will exhibit enhanced connectivity and reward-hypersensitive individuals will exhibit blunted connectivity. We further hypothesized that both enhanced/blunted corticostriatal connectivity with DMN would be associated more strongly with behavioral motivation than hedonic anhedonia. To test these hypotheses, we conducted an ROI-based network psychophysiological interaction (nPPI) analysis using the DMN as seed and VS as target. We then conducted linear regressions for the difference in connectivity for task-based contrasts targeting incentive salience (i.e., HS>LS, LG>N, Large LL>N), regressing these contrasts onto models of composite reward sensitivity (RS), Behavioral Motivation (matching the RT contrast to the PPI contrast), and their interaction (RS x Behavioral Motivation). We also tested models of RS x TEPSa and Behavioral Motivation x TEPSa.

**Figure 5.**
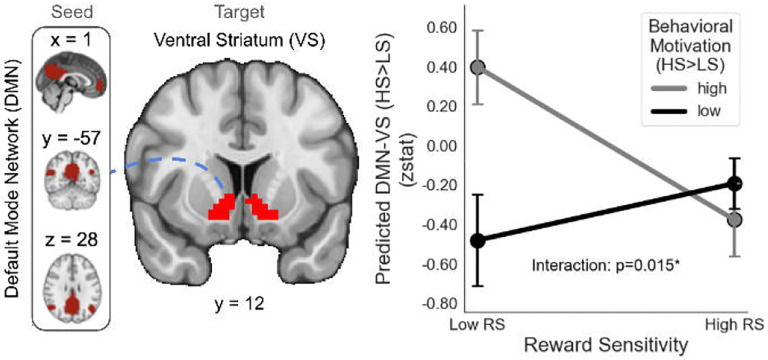
Behavioral motivation modulates the relationship between reward sensitivity (RS) and DMN-VS connectivity during reward anticipation. Interaction plot showing the effect of reward sensitivity and behavioral motivation (HS>LS) on DMN-VS connectivity (z-statistic), with predicted connectivity levels across low and high reward sensitivity (10th/90th percentiles) for low and high HS>LS groups (median-split), presented with standard error of the mean (SEM) error bars and a significant interaction (p = 0.015, annotated). For individuals with lower reward sensitivity, high behavioral motivation is associated with increased DMN-VS connectivity, while this effect is absent in those with higher reward sensitivity, highlighting a modulation of functional brain connectivity by motivational state.

Significant interactions between RS and Behavioral Motivation were observed across all three reward contexts. Specifically, HS>LS (β=−12.051154, *SE*=4.689934, *t*=−2.570, *p*=0.0138; Fig. 5) and for LG>N (β=−1.72548, *SE*=0.61087, *t*=−2.825, *p*=0.00721; Supplementary Fig. 1), high behavioral motivation was associated with a negative relationship between RS and DMN-VS connectivity. This relationship was weaker for moderate and low levels of behavioral motivation. In contrast, for LL>N (β=1.316432, *SE=*0.475776, *t*=2.767, *p*=0.00838), behavioral motivation showed no relationship with DMN-VS connectivity at high and moderate levels, while behavioral motivation for loss is associated with diminished DMN-VS connectivity (Supplementary Fig. 2). These effects are all preserved when controlling for TEPSa (HS>LS: *p*=0.0150; LG>N: *p*=0.00798; LL>N: *p*=0.00867). No significant interaction effects with TEPSa were observed.

## Discussion

This study provides new evidence that behavioral motivation moderates the relationship between trait reward sensitivity and default mode network-ventral striatum (DMN-VS) connectivity during reward anticipation. Our study revealed complex interactions between trait reward sensitivity, behavioral motivation, and neural responses during reward anticipation. Behaviorally, participants demonstrated significant modulation of reaction times based on reward salience, as indexed by HS>LS (high stakes vs. low stakes), responding faster for high-stakes trials relative to low-stakes trials (Fig. 3B). At the neural level, ventral striatum activation was significantly modulated by incentive magnitude, with highest activation observed for large gain trials. These findings persisted after controlling for anticipatory pleasure, suggesting a robust neurobiological mechanism underlying individual differences in reward processing.

These findings align with existing literature on the neural basis of reward processing, which has consistently implicated the ventral striatum as a key region in reward anticipation (Knutson et al., 2001; Haber & Knutson, 2010). Our observation that ventral striatal activation is modulated by incentive magnitude during the anticipation phase replicates previous research using the Monetary Incentive Delay task (Oldham et al., 2018; Büchel et al., 2017). Additionally, our behavioral findings showing faster reaction times for high-stakes trials are consistent with motivational theories that posit enhanced performance under conditions of greater incentive salience (Berridge & Robinson, 2016). The interaction between anhedonia and reward sensitivity in predicting HS>LS extends earlier work by demonstrating that the translation of trait-level reward sensitivity into observable motivated behavior depends critically on individual differences in anticipatory pleasure capacity.

The present study makes several novel contributions to the field of reward processing and motivation. First, we demonstrate that the relationship between reward sensitivity and corticostriatal connectivity during reward anticipation is not uniform but rather moderated by behavioral motivation, as indexed by HS>LS. Specifically, we find that for individuals with a pronounced HS>LS pattern, increasing reward sensitivity is associated with reduced connectivity between the default mode network (DMN) and the ventral striatum (VS). In contrast, this relationship is attenuated or absent for those with a less pronounced HS>LS pattern. This suggests that high behavioral motivation may facilitate a more adaptive neural balance between goal-directed reward anticipation (VS) and internally focused cognition (DMN). When motivation is high, a disengagement of self-referential thought may allow for more efficient allocation of neural resources toward reward-related cues, enhancing the ability to pursue goals. Conversely, low behavioral motivation may reflect a failure to suppress internally directed cognition during reward anticipation, potentially interfering with goal-directed action (Insel et al., 2017; Harsay et al., 2011; Murty et al., 2017).

Second, our findings highlight the importance of considering both trait (reward sensitivity) and state (behavioral motivation) factors when examining reward anticipation. Prior studies often have treated reward sensitivity as a singular predictor of reward-related neural activity. However, our results suggest that its effects on corticostriatal connectivity are contingent upon motivational state, highlighting the need for an integrative approach. Notably, although reward sensitivity is typically linked to responsiveness to gains, prospect theory (Kahneman & Tversky, 1979) suggests that losses can exert an equally—if not more—powerful influence on decision-making and motivation. This aligns with evidence from Pizzagalli et al. (2005), which indicates that dysfunction in loss-based reward processing can significantly impact motivational engagement. By accounting for both stable individual differences and dynamic fluctuations in motivation—including responses to both gains and losses—we provide a more nuanced understanding of how reward processing varies across individuals. This perspective may help reconcile discrepancies in previous research, particularly studies that have reported mixed findings regarding the relationship between reward sensitivity and striatal engagement. Studies that do not account for motivational state and loss-related influences may overlook key moderating factors, leading to inconsistent conclusions about the role of reward sensitivity in neural processing.

Finally, these results contribute to a growing body of literature suggesting that DMN-VS connectivity plays a critical role in motivational disorders. Our findings suggest that in highly reward-sensitive, highly motivated individuals, reduced DMN-VS connectivity may reflect an adaptive suppression of self-referential processes during reward anticipation, driven by incentive salience, allowing for more effective engagement with external reward cues. However, in individuals with lower reward sensitivity or motivation, persistent DMN engagement may interfere with goal-directed motivation, potentially leading to maladaptive patterns of reward processing. This dynamic has relevance for conditions such as depression, where excessive DMN activity has been implicated in rumination and reduced motivation (Di Martino et al., 2008). In depressive states, persistent engagement of self-referential thought during reward anticipation may impair the ability to mobilize effort toward potential rewards, contributing to symptoms of anhedonia and amotivation (Whitton et al., 2015; Treadway & Zald, 2011). Beyond the DMN, however, the VS also interacts with the executive control network (ECN) to support goal-directed behavior. Murty et al. (2017) highlight how striatal-executive control interactions help translate reward anticipation into adaptive action, a process that may be particularly relevant for understanding motivational deficits. Given these findings, future research should further investigate whether disruptions in both DMN-VS and ECN-VS connectivity serve as neurobiological mechanisms (Treadway, 2015) underlying motivational impairments in disorders such as anhedonia, depression, or apathy-related syndromes (Roiser & Husain, 2023).

Several limitations of the current study warrant consideration. First, our sample size (N=48) was limited due to constraints imposed by the COVID-19 pandemic, which necessitated early termination of data collection. Replication in a larger, more diverse sample would strengthen the generalizability of our findings. Second, our sample was predominantly female (77.3%), which may limit the applicability of our findings to males, especially given evidence of sex differences in reward processing (Dreher et al., 2007). Future studies should include gender as a covariate and ensure more balanced representation. Third, although the MID task effectively captures anticipatory processes, it represents just one paradigm for studying reward processing. Multiple components of reward processing (anticipation, consumption, learning) engage partially overlapping but distinct neural circuits (Wang et al., 2016; Haber & Knutson, 2010). Similarly, we observed different patterns of connectivity for gain versus loss anticipation, suggesting distinct neural mechanisms for approach versus avoidance motivation (Supplementary Figures 1 and 2; e.g., Aupperle et al., 2015; Spielberg et al., 2012). Future research should employ multiple task paradigms to dissociate these processes more clearly. Finally, understanding how these findings relate to problematic substance use remains an important avenue for future research, as altered reward sensitivity and motivation have been implicated in addiction vulnerability (Volkow et al., 2010).

Our findings underscore the importance of considering individual differences in both trait reward sensitivity and state-level behavioral motivation when investigating the neural correlates of reward anticipation. The significant moderation of DMN-VS connectivity by behavioral motivation suggests that the neural mechanisms underlying reward processing are not static but dynamically adjust based on context and individual characteristics. Here, we can find direct implications for clinical conditions characterized by disrupted reward processing, such as depression and addiction. For example, in depression, deficits in anticipatory pleasure and motivation are hallmark symptoms (Treadway & Zald, 2011), and our results suggest that these deficits may be linked to altered connectivity between the default mode network (DMN) and the ventral striatum (VS). Specifically, diminished motivational states could lead to reduced DMN-VS connectivity, impairing the brain’s ability to anticipate and pursue rewarding outcomes (Volkow et al., 2010). This neural disruption may underlie the blunted motivation and anhedonia commonly seen in depression, highlighting the importance of motivational context.

Furthermore, in addiction, where dysregulated reward processing plays a key role, our findings suggest that disrupted DMN-VS connectivity could contribute to impairments in decision-making related to rewards. Addicts often show altered brain activity when anticipating rewards, which may stem from dysfunctional interactions between the DMN and VS, regions involved in goal-directed behavior and self-regulation. Interpreting the influence of connectivity between motivation and individual reward sensitivity could lead to more targeted interventions that address neural disruptions in addiction.

In conclusion, our study highlights the value of integrating behavioral, self-report, and neuroimaging measures to develop a more comprehensive model of reward processing. This approach may help us better understand the complex interplay between stable traits (such as reward sensitivity) and context-dependent states (such as current levels of motivation) in clinical populations (Pizzagalli, 2014). Ultimately, these insights may inform more personalized treatments for conditions like depression and addiction, where reward dysfunction is a central feature, by tailoring interventions based on an individual’s specific neural and motivational profiles.

## Supplementary Material

Supplement 1

Supplement 2

Supplement 3

Supplement 4

Supplement 5

## Figures and Tables

**Figure 1.**
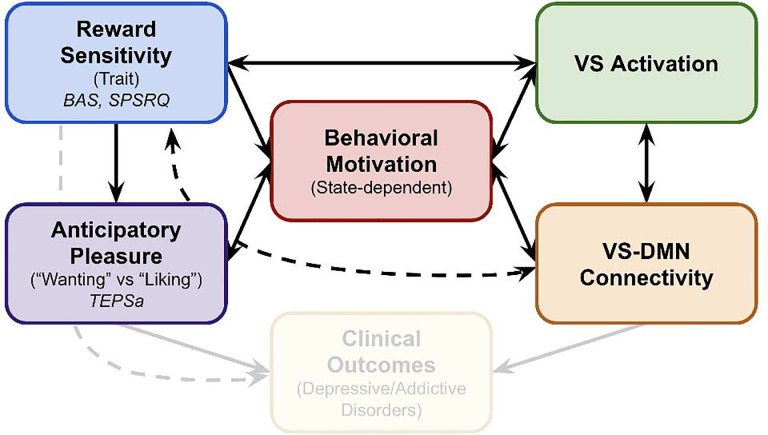
Conceptual model of reward processing and neural mechanisms. This model illustrates hypothesized relationships between reward sensitivity (RS), anticipatory pleasure (TEPSa), behavioral motivation, and related neural mechanisms. RS influences VS activation during reward anticipation and contributes to anticipatory pleasure. Behavioral motivation serves as a state-dependent mediator that enhances VS activation and moderates VS-DMN connectivity patterns. The model proposes that aberrant RS (either hypo- or hyper-sensitivity) leads to distinct neural signatures that could have implications for clinical presentations, with VS-DMN connectivity potentially serving as a mechanistic pathway linking individual differences in reward processing to anhedonia or impulsivity. Solid arrows represent direct relationships, whereas dashed lines indicate more complex pathways that may involve additional factors. Clinical outcomes (shown with lower contrast) represent potential downstream effects not directly tested in the current study.

## References

[R1] Alloy L. B., Abramson L. Y., Walshaw P. D., Cogswell A., Smith J. M., Neeren A. M., Hughes M. E., Iacoviello B. M., Gerstein R. K., Keyser J., Urosevic S., & Nusslock R. (2006). Behavioral Approach System (BAS) Sensitivity and Bipolar Spectrum Disorders: A Retrospective and Concurrent Behavioral High-Risk Design. Motivation and Emotion, 30(2), 143–155. 10.1007/s11031-006-9003-3

[R2] Alloy L. B., Abramson L. Y., Walshaw P. D., Gerstein R. K., Keyser J. D., Whitehouse W. G., Urosevic S., Nusslock R., Hogan M. E., & Harmon-Jones E. (2009). Behavioral approach system (BAS)–relevant cognitive styles and bipolar spectrum disorders: Concurrent and prospective associations. Journal of Abnormal Psychology, 118(3), 459–471. 10.1037/a0016604 19685944 PMC2761740

[R3] Alloy L. B., Bender R. E., Whitehouse W. G., Wagner C. A., Liu R. T., Grant D. A., Jager-Hyman S., Molz A., Choi J. Y., Harmon-Jones E., & Abramson L. Y. (2012). High Behavioral Approach System (BAS) sensitivity, reward responsiveness, and goal-striving predict first onset of bipolar spectrum disorders: A prospective behavioral high-risk design. Journal of Abnormal Psychology, 121(2), 339–351. 10.1037/a0025877 22004113 PMC3403678

[R4] Alloy L. B., Olino T., Freed R. D., & Nusslock R. (2016). Role of Reward Sensitivity and Processing in Major Depressive and Bipolar Spectrum Disorders. Behavior Therapy, 47(5), 600–621. 10.1016/j.beth.2016.02.014 27816074 PMC5119651

[R5] Aupperle R. L., Melrose A. J., Francisco A., Paulus M. P., & Stein M. B. (2015). Neural substrates of approach-avoidance conflict decision-making. Human Brain Mapping, 36(2), 449–462. 10.1002/hbm.22639 25224633 PMC4300249

[R6] Berridge K. C., & Robinson T. E. (2016). Liking, wanting, and the incentive-sensitization theory of addiction. American Psychologist, 71(8), 670–679. 10.1037/amp0000059 27977239 PMC5171207

[R7] Bretzke M., Wahl H., Plichta M. M., Wolff N., Roessner V., Vetter N. C., & Buse J. (2021). Ventral Striatal Activation During Reward Anticipation of Different Reward Probabilities in Adolescents and Adults. Frontiers in Human Neuroscience, 15. 10.3389/fnhum.2021.649724 PMC8093817 33958995

[R8] Büchel C., Peters J., Banaschewski T., Bokde A. L. W., Bromberg U., Conrod P. J., Flor H., Papadopoulos D., Garavan H., Gowland P., Heinz A., Walter H., Ittermann B., Mann K., Martinot J.-L., Paillère-Martinot M.-L., Nees F., Paus T., Pausova Z., … Knutson B. (2017). Blunted ventral striatal responses to anticipated rewards foreshadow problematic drug use in novelty-seeking adolescents. Nature Communications, 8(1), 14140. 10.1038/ncomms14140 PMC5321762 28221370

[R9] Capa R. L., & Bouquet C. A. (2018). Individual Differences in Reward Sensitivity Modulate the Distinctive Effects of Conscious and Unconscious Rewards on Executive Performance. Frontiers in Psychology, 9. 10.3389/fpsyg.2018.00148 PMC5820315 29503624

[R10] Cardoso Melo R. D., Groen R. N., & Hartman C. A. (2022). Reward Sensitivity at Age 13 Predicts the Future Course of Psychopathology Symptoms. Frontiers in Psychiatry, 13. 10.3389/fpsyt.2022.818047 PMC8962629 35360134

[R11] Cardoso Melo R. D., Schreuder M. J., Groen R. N., Sarsembayeva D., & Hartman C. A. (2023). Reward sensitivity across the lifespan in males and females and its associations with psychopathology. Personality and Individual Differences, 204, 112041. 10.1016/j.paid.2022.112041

[R12] Carruzzo F., Giarratana A. O., del Puppo L., Kaiser S., Tobler P. N., & Kaliuzhna M. (2023). Neural bases of reward anticipation in healthy individuals with low, mid, and high levels of schizotypy. Scientific Reports, 13(1), Article 1. 10.1038/s41598-023-37103-2 PMC10279672 37337085

[R13] Carter R. M., MacInnes J. J., Huettel S. A., & Adcock R. A. (2009). Activation in the VTA and nucleus accumbens increases in anticipation of both gains and losses. Frontiers in Behavioral Neuroscience, 3. 10.3389/neuro.08.021.2009 PMC2742668 19753142

[R14] Carver C. S., & White T. L. (1994). Behavioral inhibition, behavioral activation, and affective responses to impending reward and punishment: The BIS/BAS Scales. Journal of Personality and Social Psychology, 67(2), 319–333. 10.1037/0022-3514.67.2.319

[R15] Chat I. K.-Y., Dunning E. E., Bart C. P., Carroll A. L., Grehl M. M., Damme K. S., Abramson L. Y., Nusslock R., & Alloy L. B. (2022). The Interplay Between Reward-Relevant Life Events and Trait Reward Sensitivity in Neural Responses to Reward Cues. Clinical Psychological Science, 10(5), 869–884. 36381350 10.1177/21677026211056627 PMC9662616

[R16] Cooper J. C., & Knutson B. (2008). Valence and salience contribute to nucleus accumbens activation. NeuroImage, 39(1), 538–547. 10.1016/j.neuroimage.2007.08.009 17904386 PMC2169259

[R17] Daniels A., Wellan S. A., Beck A., Erk S., Wackerhagen C., Romanczuk-Seiferth N., Schwarz K., Schweiger J. I., Meyer-Lindenberg A., Heinz A., & Walter H. (2025). Anhedonia relates to reduced striatal reward anticipation in depression but not in schizophrenia or bipolar disorder: A transdiagnostic study. Cognitive, Affective, & Behavioral Neuroscience. 10.3758/s13415-024-01261-1 PMC11906564 39885092

[R18] Davis C., Patte K., Levitan R., Reid C., Tweed S., & Curtis C. (2007). From motivation to behaviour: A model of reward sensitivity, overeating, and food preferences in the risk profile for obesity. Appetite, 48(1), 12–19. 10.1016/j.appet.2006.05.016 16875757

[R19] Dobryakova E., & Smith D. V. (2021). Reward Enhances Connectivity between the Ventral Striatum and the Default Mode Network [Preprint]. Neuroscience. 10.1101/2021.07.28.454086 PMC9343171 35724856

[R20] Dreher J.-C., Schmidt P. J., Kohn P., Furman D., Rubinow D., & Berman K. F. (2007). Menstrual cycle phase modulates reward-related neural function in women. Proceedings of the National Academy of Sciences of the United States of America, 104(7), 2465–2470. 10.1073/pnas.0605569104 17267613 PMC1892961

[R21] Duffy E. (1957). The psychological significance of the concept of “arousal” or “activation.” Psychological Review, 64(5), 265–275. 10.1037/h0048837 13494613

[R22] Esteban O., Markiewicz C. J., Blair R. W., Moodie C. A., Isik A. I., Erramuzpe A., Kent J. D., Goncalves M., DuPre E., Snyder M., Oya H., Ghosh S. S., Wright J., Durnez J., Poldrack R. A., & Gorgolewski K. J. (2019). fMRIPrep: A robust preprocessing pipeline for functional MRI. Nature Methods, 16(1), Article 1. 10.1038/s41592-018-0235-4 PMC6319393 30532080

[R23] Fareri D. S., Hackett K., Tepfer L. J., Kelly V., Henninger N., Reeck C., Giovannetti T., & Smith D. V. (2021). Age-Related Differences in Ventral Striatal and Default Mode Network Function During Reciprocated Trust [Preprint]. Neuroscience. 10.1101/2021.07.29.454071 PMC9308012 35504565

[R24] Fellows L. K. (2004). The Cognitive Neuroscience of Human Decision Making: A Review and Conceptual Framework. Behavioral and Cognitive Neuroscience Reviews, 3(3), 159–172. 10.1177/1534582304273251 15653813

[R25] Filimon F., Nelson J. D., Sejnowski T. J., Sereno M. I., & Cottrell G. W. (2020). The ventral striatum dissociates information expectation, reward anticipation, and reward receipt. Proceedings of the National Academy of Sciences, 117(26), 15200–15208. 10.1073/pnas.1911778117 PMC7334472 32527855

[R26] Friston K. J., Buechel C., Fink G. R., Morris J., Rolls E., & Dolan R. J. (1997). Psychophysiological and Modulatory Interactions in Neuroimaging. NeuroImage, 6(3), 218–229. 10.1006/nimg.1997.0291 9344826

[R27] Gaher R. M., Hahn A. M., Shishido H., Simons J. S., & Gaster S. (2015). Associations between sensitivity to punishment, sensitivity to reward, and gambling. Addictive Behaviors, 42, 180–184. 10.1016/j.addbeh.2014.11.014 25481451

[R28] Gard D. E., Gard M. G., Kring A. M., & John O. P. (2006). Anticipatory and consummatory components of the experience of pleasure: A scale development study. Journal of Research in Personality, 40(6), 1086–1102. 10.1016/j.jrp.2005.11.001

[R29] Goldstein R. Z., Alia-Klein N., Tomasi D., Zhang L., Cottone L. A., Maloney T., Telang F., Caparelli E. C., Chang L., Ernst T., Samaras D., Squires N. K., & Volkow N. D. (2007). Is Decreased Prefrontal Cortical Sensitivity to Monetary Reward Associated With Impaired Motivation and Self-Control in Cocaine Addiction? American Journal of Psychiatry, 164(1), 43–51. 10.1176/ajp.2007.164.1.43 17202543 PMC2435056

[R30] Gorgolewski K., Burns C. D., Madison C., Clark D., Halchenko Y. O., Waskom M. L., & Ghosh S. S. (2011). Nipype: A Flexible, Lightweight and Extensible Neuroimaging Data Processing Framework in Python. Frontiers in Neuroinformatics, 5. 10.3389/fninf.2011.00013 PMC3159964 21897815

[R31] Gorgolewski K. J., Auer T., Calhoun V. D., Craddock R. C., Das S., Duff E. P., Flandin G., Ghosh S. S., Glatard T., Halchenko Y. O., Handwerker D. A., Hanke M., Keator D., Li X., Michael Z., Maumet C., Nichols B. N., Nichols T. E., Pellman J., … Poldrack R. A. (2016). The brain imaging data structure, a format for organizing and describing outputs of neuroimaging experiments. Scientific Data, 3(1), 160044. 10.1038/sdata.2016.44 27326542 PMC4978148

[R32] Gray J. A. (1987). The neuropsychology of emotion and personality. In Cognitive neurochemistry (pp. 171–190). Oxford University Press.

[R33] Grill F., Nyberg L., & Rieckmann A. (2021). Neural correlates of reward processing: Functional dissociation of two components within the ventral striatum. Brain and Behavior, 11(2), e01987. 10.1002/brb3.1987 33300306 PMC7882172

[R34] Haber S. N., & Knutson B. (2010). The Reward Circuit: Linking Primate Anatomy and Human Imaging. Neuropsychopharmacology, 35(1), 4–26. 10.1038/npp.2009.129 19812543 PMC3055449

[R35] Halchenko Y. O., Goncalves M., Ghosh S., Velasco P., Visconti di Oleggio Castello M., Salo T., Wodder J. T., Hanke M., Sadil P., Gorgolewski K. J., Ioanas H.-I., Rorden C., Hendrickson T. J., Dayan M., Houlihan S. D., Kent J., Strauss T., Lee J., To I., … Kennedy D. N. (2024). HeuDiConv—Flexible DICOM conversion into structured directory layouts. Journal of Open Source Software, 9(99), 5839. 10.21105/joss.05839 39323511 PMC11423922

[R36] Hull C. L. (1943). Principles of behavior: An introduction to behavior theory (pp. x, 422). Appleton-Century.

[R37] Jenkinson M., Beckmann C. F., Behrens T. E. J., Woolrich M. W., & Smith S. M. (2012). FSL. NeuroImage, 62(2), 782–790. 10.1016/j.neuroimage.2011.09.015 21979382

[R38] Jorm A. F., Christensen H., Henderson A. S., Jacomb P. A., Korten A. E., & Rodgers B. (1998). Using the BIS/BAS scales to measure behavioural inhibition and behavioural activation: Factor structure, validity and norms in a large community sample. Personality and Individual Differences, 26(1), 49–58. 10.1016/S0191-8869(98)00143-3

[R39] Kahneman D., & Tversky A. (1979). Prospect Theory: An Analysis of Decisions under Risk. Econometrica, 47(2), 263–292.

[R40] Knutson B., Adams C. M., Fong G. W., & Hommer D. (2001). Anticipation of Increasing Monetary Reward Selectively Recruits Nucleus Accumbens. The Journal of Neuroscience, 21(16). 10.1523/JNEUROSCI.21-16-j0002.2001 PMC6763187 11459880

[R41] Nickerson L. D., Smith S. M., Öngür D., & Beckmann C. F. (2017). Using Dual Regression to Investigate Network Shape and Amplitude in Functional Connectivity Analyses. Frontiers in Neuroscience, 11. 10.3389/fnins.2017.00115 PMC5346569 28348512

[R42] Oka T., Sasaki A., & Kobayashi N. (2024). A Transdiagnostic Dimensional Approach to Behavioral Dysregulation: Examining Reward and Punishment Sensitivity Across Psychopathology (p. 2024.10.14.24315505). medRxiv. 10.1101/2024.10.14.24315505 40441645

[R43] Oldham S., Murawski C., Fornito A., Youssef G., Yücel M., & Lorenzetti V. (2018). The anticipation and outcome phases of reward and loss processing: A neuroimaging meta-analysis of the monetary incentive delay task. Human Brain Mapping, 39(8), 3398–3418. 10.1002/hbm.24184 29696725 PMC6055646

[R44] O’Reilly J. X., Woolrich M. W., Behrens T. E. J., Smith S. M., & Johansen-Berg H. (2012). Tools of the trade: Psychophysiological interactions and functional connectivity. Social Cognitive and Affective Neuroscience, 7(5), 604–609. 10.1093/scan/nss055 22569188 PMC3375893

[R45] Pessoa L. (2015). Multiple influences of reward on perception and attention. Visual Cognition, 23(1–2), 272–290. 10.1080/13506285.2014.974729 26190929 PMC4503337

[R46] Posner J., Cha J., Wang Z., Talati A., Warner V., Gerber A., Peterson B. S., & Weissman M. (2016). Increased Default Mode Network Connectivity in Individuals at High Familial Risk for Depression. Neuropsychopharmacology, 41(7), 1759–1767. 10.1038/npp.2015.342 26593265 PMC4869043

[R47] Potsch L., & Rief W. (2023). Transdiagnostic considerations of the relationship between reward sensitivity and psychopathological symptoms—A cross-lagged panel analysis. BMC Psychiatry, 23(1), 650. 10.1186/s12888-023-05139-3 37667190 PMC10478275

[R48] Rosell-Negre P., Bustamante J. C., Fuentes-Claramonte P., Costumero V., Benabarre S., & Barrós-Loscertales A. (2017). Monetary reward magnitude effects on behavior and brain function during goal-directed behavior. Brain Imaging and Behavior, 11(4), 1037–1049. 10.1007/s11682-016-9577-7 27473167

[R49] Saris I. M. J., Penninx B. W. J. H., Dinga R., van Tol M.-J., Veltman D. J., van der Wee N. J. A., & Aghajani M. (2020). Default Mode Network Connectivity and Social Dysfunction in Major Depressive Disorder. Scientific Reports, 10(1), 194. 10.1038/s41598-019-57033-2 31932627 PMC6957534

[R50] Smith D. V. (2016). Toward a cumulative science of functional integration: A meta-analysis of psychophysiological interactions. 10.1002/hbm.23216 PMC4945436 27145472

[R51] Smith D. V., Clithero J. A., Boltuck S. E., & Huettel S. A. (2014). Functional connectivity with ventromedial prefrontal cortex reflects subjective value for social rewards. Social Cognitive and Affective Neuroscience, 9(12), 2017–2025. 10.1093/scan/nsu005 24493836 PMC4249475

[R52] Smith D. V., & Delgado M. (2017). Meta-analysis of psychophysiological interactions: Revisiting cluster-level thresholding and sample sizes. Human Brain Mapping, 38(1), 588–591. 10.1002/hbm.23354 27543687 PMC5148685

[R53] Smith D. V., Wyngaarden J., Sharp C. J., Sazhin D., Zaff O., Fareri D., & Jarcho J. (2024). An fMRI dataset of social and nonsocial reward processing in young adults. Data in Brief, 53, 110197. 10.1016/j.dib.2024.110197 38406247 PMC10885710

[R54] Smith S. M., Fox P. T., Miller K. L., Glahn D. C., Fox P. M., Mackay C. E., Filippini N., Watkins K. E., Toro R., Laird A. R., & Beckmann C. F. (2009). Correspondence of the brain’s functional architecture during activation and rest. Proceedings of the National Academy of Sciences, 106(31), 13040–13045. 10.1073/pnas.0905267106 PMC2722273 19620724

[R55] Smith S. M., Jenkinson M., Woolrich M. W., Beckmann C. F., Behrens T. E. J., Johansen-Berg H., Bannister P. R., De Luca M., Drobnjak I., Flitney D. E., Niazy R. K., Saunders J., Vickers J., Zhang Y., De Stefano N., Brady J. M., & Matthews P. M. (2004). Advances in functional and structural MR image analysis and implementation as FSL. NeuroImage, 23, S208–S219. 10.1016/j.neuroimage.2004.07.051 15501092

[R56] Spielberg J. M., Miller G. A., Warren S. L., Engels A. S., Crocker L. D., Banich M. T., Sutton B. P., & Heller W. (2012). A brain network instantiating approach and avoidance motivation. Psychophysiology, 49(9), 1200–1214. 10.1111/j.1469-8986.2012.01443.x 22845892 PMC4559331

[R57] Torrubia R., Ávila C., Moltó J., & Caseras X. (2001). The Sensitivity to Punishment and Sensitivity to Reward Questionnaire (SPSRQ) as a measure of Gray’s anxiety and impulsivity dimensions. Personality and Individual Differences, 31(6), 837–862. 10.1016/S0191-8869(00)00183-5

[R58] Tziortzi A. C., Searle G. E., Tzimopoulou S., Salinas C., Beaver J. D., Jenkinson M., Laruelle M., Rabiner E. A., & Gunn R. N. (2011). Imaging dopamine receptors in humans with [11C]-(+)-PHNO: Dissection of D3 signal and anatomy. NeuroImage, 54(1), 264–277. 10.1016/j.neuroimage.2010.06.044 20600980

[R59] Utevsky A. V., Smith D. V., Young J. S., & Huettel S. A. (2017). Large-Scale Network Coupling with the Fusiform Cortex Facilitates Future Social Motivation. eNeuro, 4(5), ENEURO.0084–17.2017. 10.1523/ENEURO.0084-17.2017 PMC5635486 29034316

[R60] Veldhoven D. T., Roozen H., & Vingerhoets A. (2020). The Association between Reward Sensitivity and Activity Engagement: The Influence of Delay Discounting and Anhedonia. Alcohol and Alcoholism (Oxford, Oxfordshire), 55(2), 215–224. 10.1093/alcalc/agz105 31998950 PMC7082492

[R61] Volkow N. D., Wang G.-J., Fowler J. S., Logan J., Jayne M., Franceschi D., Wong C., Gatley S. J., Gifford A. N., Ding Y.-S., & Pappas N. (2002). “Nonhedonic” food motivation in humans involves dopamine in the dorsal striatum and methylphenidate amplifies this effect. Synapse, 44(3), 175–180. 10.1002/syn.10075 11954049

[R62] Volkow N. D., Wang G.-J., Fowler J. S., Tomasi D., Telang F., & Baler R. (2010). Addiction: Decreased reward sensitivity and increased expectation sensitivity conspire to overwhelm the brain’s control circuit. BioEssays, 32(9), 748–755. 10.1002/bies.201000042 20730946 PMC2948245

[R63] Wang K. S., Smith D. V., & Delgado M. R. (2016). Using fMRI to study reward processing in humans: Past, present, and future. Journal of Neurophysiology, 115(3), 1664–1678. 10.1152/jn.00333.2015 26740530 PMC4808130

[R64] Woolrich M. W., Ripley B. D., Brady M., & Smith S. M. (2001). Temporal Autocorrelation in Univariate Linear Modeling of FMRI Data. NeuroImage, 14(6), 1370–1386. 10.1006/nimg.2001.0931 11707093

[R65] Wyngaarden J. B., Johnston C. R., Sazhin D., Dennison J. B., Zaff O., Fareri D., McCloskey M., Alloy L. B., Smith D. V., & Jarcho J. M. (2024). Corticostriatal responses to social reward are linked to trait reward sensitivity and subclinical substance use in young adults. Social Cognitive and Affective Neuroscience, 19(1), nsae033. 10.1093/scan/nsae033 38779870 PMC11182064

