# Supplementary material for: Trait reward sensitivity and behavioral motivation shape connectivity between the default mode network and the striatum during reward anticipation
Source: bioRxiv. 2025 May 7:2025.04.17.649386. Preprint. [Version 2] doi: 10.1101/2025.04.17.649386 (PMC12247891; doi:10.1101/2025.04.17.649386)
Supplement: Supplementary file 1 [file media-1.docx]

**Supplementary Methods.** ***Deviations from Pre-Registration.***

Whereas our pre-registration outlined an analysis of reaction time (RT) patterns across trial types to assess behavioral motivation, we later expanded our approach to include specific contrasts for large gain > neutral (LG > N) and large loss > neutral (LL > N). These contrasts were added to more directly capture the impact of reward and loss outcomes on behavioral motivation, given prior evidence that both large rewards and large losses significantly influence motivation (Kahneman & Tversky, 1979). Although these contrasts were not pre-registered, they were deemed essential for a more precise analysis of reward salience and were fully aligned with the study's core aims.
