# Supplementary material for: Trait reward sensitivity and behavioral motivation shape connectivity between the default mode network and the striatum during reward anticipation
Source: bioRxiv. 2025 May 7:2025.04.17.649386. Preprint. [Version 2] doi: 10.1101/2025.04.17.649386 (PMC12247891; doi:10.1101/2025.04.17.649386)
Supplement: Supplementary file 2 [file media-2.docx]

**Supplementary Table 1.** ***Pairwise comparisons of reaction times across trial conditions.***

| Contrast | Estimate | *t* | *p* |
| --- | --- | --- | --- |
| Large Gain - Small Gain | -0.007629 | -1.278 | 0.705 |
| Large Gain - Neutral | -0.02104 | -3.525 | 0.0048** |
| Large Gain - Small Loss | -0.016521 | -2.768 | 0.0484* |
| Large Gain - Large Loss | -0.000424 | -0.071 | 1 |
| Small Gain - Neutral | -0.013411 | -2.247 | 0.1675 |
| Small Gain - Small Loss | -0.008892 | -1.49 | 0.5705 |
| Small Gain - Large Loss | 0.007205 | 1.207 | 0.7473 |
| Neutral - Small Loss | 0.004519 | 0.757 | 0.9424 |
| Neutral - Large Loss | 0.020616 | 3.454 | 0.0061** |
| Small Loss - Large Loss | 0.016098 | 2.697 | 0.0584 |
| *(p<.05); **(p<.01). |  |  |  |
