# Supplementary material for: Trait reward sensitivity and behavioral motivation shape connectivity between the default mode network and the striatum during reward anticipation
Source: bioRxiv. 2025 May 7:2025.04.17.649386. Preprint. [Version 2] doi: 10.1101/2025.04.17.649386 (PMC12247891; doi:10.1101/2025.04.17.649386)
Supplement: Supplementary file 3 [file media-3.docx]

**Supplementary Table 2.** ***Pairwise comparisons of striatal response to incentive magnitude.*** 

| Contrast | Estimate | *t* | *p* |
| --- | --- | --- | --- |
| Large Gain - Small Gain | 0.216 | 1.996 | 0.272 |
| Large Gain - Neutral | 1.121 | 10.348 | <.0001*** |
| Large Gain - Small Loss | 0.356 | 3.288 | 0.0105* |
| Large Gain - Large Loss | 0.308 | 2.845 | 0.0393* |
| Small Gain - Neutral | 0.905 | 8.351 | <.0001*** |
| Small Gain - Small Loss | 0.14 | 1.292 | 0.6966 |
| Small Gain - Large Loss | 0.092 | 0.849 | 0.9149 |
| Neutral - Small Loss | -0.765 | -7.06 | <.0001*** |
| Neutral - Large Loss | -0.813 | -7.503 | <.0001*** |
| Small Loss - Large Loss | -0.048 | -0.443 | 0.992 |
| *(p<.05); ***(p<.001). |  |  |  |
