# Supplementary material for: Trait reward sensitivity and behavioral motivation shape connectivity between the default mode network and the striatum during reward anticipation
Source: bioRxiv. 2025 May 7:2025.04.17.649386. Preprint. [Version 2] doi: 10.1101/2025.04.17.649386 (PMC12247891; doi:10.1101/2025.04.17.649386)
Supplement: Supplementary file 4 [file media-4.docx]

**Supplementary Figure 1.** ***Behavioral motivation (LG>N) modulates the relationship between reward sensitivity (RS) and DMN-VS connectivity during reward anticipation.***
